# Efficiency of the Lausanne Clinical Pathway for Proximal Femoral Fractures

**DOI:** 10.3389/fsurg.2015.00005

**Published:** 2015-02-19

**Authors:** Nicole Fleury, François Chevalley, Eve Rubli, Pauline Coti, Alain Farron, Brigitte M. Jolles

**Affiliations:** ^1^Centre Hospitalier Universitaire Vaudois (CHUV) and University of Lausanne, Lausanne, Switzerland

**Keywords:** clinical pathway, hip fractures, surgery efficiency, proximal femoral fracture, elderly patients

## Abstract

**Purpose/Introduction:** The number of hip fractures is rising, due to increases in life expectancy. In such cases, patients are at risk from post-operative complications and subsequently the average length of hospitalization may be extended. In 2011, we established a clinical pathway (CP), a specific model of care for patient-care management, to improve the clinical and economic outcomes of proximal femoral fracture management in elderly patients. The goal was to evaluate the CP using clinical, process, and financial indicators.

**Methods:** We included all surgical patients aged 65 and over, admitted to the emergency department with a fracture of the proximal femur following a fall. Assessment parameters included three performance indicators: clinical, process, and financial. The clinical indicators were the presence or absence of acute delirium on the third post-operative day, diagnosis of nosocomial pneumonia, and the number of patients fulfilling at least 75% of their nutritional requirements at the end of the hospitalization period. The process indicator was the time interval between arrival at the emergency department and surgery. The financial indicator was based on the number of days spent in hospital.

**Results:** From 2011 to 2013, 669 patients were included in the CP. We observed that the average length of stay in hospital decreased as soon as the CP was implemented and stabilized afterwards. The goal of 90% of patients undergoing surgery within 48 h of arrival in the emergency department was surpassed in 2013 (93.1%). Furthermore, we observed an improvement in the clinical indicators.

**Conclusion:** The application of a CP allowed an improvement in the qualitative and quantitative efficiency of proximal femoral fracture management in elderly patients, in terms of clinical, process, and financial factors.

## Introduction

Due to the increase in life expectancy, and the rise in the average age of the population, the number of hip fractures is still growing. According to Gullberg, the projected annual incidence of hip fractures, world-wide, will rise from 1.26 million in 1990 to 2.6 million by the year 2025 and to 4.5 million by 2050 (1). In our university hospital, over 200 patients annually, present with fractures of the proximal part of the femur, which require extended mean periods of hospitalization, subsequently resulting in elevated medical costs. Many of these patients suffer from undernutrition (2) and post-operative delirium (3), which can lead to post-operative complications, institutionalization, or death. Also, it was found that an operative delay of 2 days and more, after hospital admission, was associated with increased mortality (4). With early detection and treatment, post-operative morbidity and mortality rates could be reduced. Traditionally, clinicians looking after patients work individually, and not necessarily in a coordinated manner. Taking into consideration these different points, a clinical pathway (CP) was established in 2011, to improve the qualitative and quantitative efficiency of proximal femoral fracture management in elderly patients (5). Some publications (6–8) show encouraging results using various CP programs. This CP, founded on evidence-based medicine guidelines, proposed to establish a system of patient-care management, for a specific patient population, involving a multidisciplinary team (9).This created interdisciplinary associations between emergency physicians, surgeons, nurses, unit management, responsible for the flow of patients through the system, physiotherapists, geriatricians, specialists in osteoporosis, and nutritionists. We evaluated this proximal femoral fracture CP, using clinical, process, and financial indicators.

## Materials and Methods

### Patients demographics

In the first instance, we included all surgical patients aged 65 and over, admitted to the emergency department with a fracture of the proximal femur following a fall. From 01.03.2011 to 31.12.2013, 669 patients were included. The men age of the cohort was 83.8 years in 2011, 83.7 years in 2012, and 82.9 years in 2013, of which 148 were men (22.1%) and 521 were women (77.9%).

### Inclusion and exclusion criteria

We excluded patients with periprosthetic fractures and pathological fractures and all polytrauma cases. Any patient that remained in the intensive care unit post-inclusion in the CP or was transferred to another unit for more than 2 days was excluded.

### Flowchart

**Figure 1:**
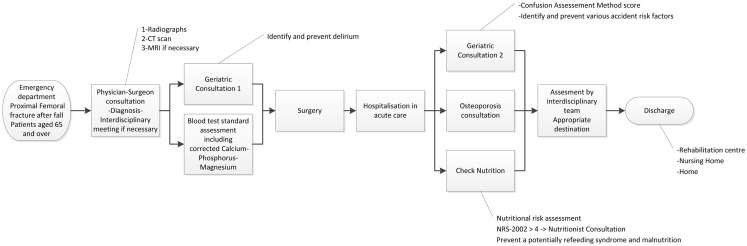
**Flowchart: clinical pathway for surgical patients aged 65 and over with a proximal femoral fracture following a fall**.

Initially, anterior–posterior pelvic and axial view radiographs of the proximal femoral fractures were performed, and were followed, when necessary, by a CT scan. MRI was only used in cases without a definitive diagnosis (Figure 1). Patients had a complete blood count, and TP, PTT, Na^+^, K^+^, glycemia, creatinine, eGFR, CK, albumin, blood group, phosphorus, magnesium, and corrected calcium blood tests, followed by a geriatric consultation in order to detect, treat, or prevent delirium incidences, where necessary. A second geriatric consultation, intended to identify and prevent various accident risk factors, was organized, to assess cognitive impairment, review medication in particular psychotropic drugs, advise on the use of walking aids, and make recommendations for the general practitioner.

The patient was transferred directly to the operating room or to the orthopedic unit pending an intervention. After surgery, each patient was monitored daily, to identify, treat, and prevent any incidences of delirium. Nutritional risk assessments were performed using the NRS-2002 method (10) on the first post-operative day and repeated weekly. Patients with an NRS-2002 ≥4 were assessed by a nutritionist in order to define nutritional requirements, to treat malnutrition, and to prevent a potentially refeeding syndrome (2).

Within 24–48 h after surgery, patients had a bedside osteoporosis consultation, in order to identify cases of osteoporotic disease and develop appropriate treatments.

During hospitalization, depending on the functional, medical, and social statuses of each patient, the appropriate destination after acute care was assessed by the interdisciplinary team. Patients could be directed to a rehabilitation center, their own homes, or a nursing home following a few days of acute rehabilitation.

### Indicators

In terms of assessment parameters, the first *clinical indicator* chosen was a positive delirium diagnosis on the third post-operative day (yes/no) based on the confusion assessment method score (CAM) (11). The presence of a nosocomial pneumonia (yes/no), the second clinical indicator, was defined by a positive AP chest radiograph or CT and antibiotherapy. The third clinical indicator was the rate of patients fulfilling at least 75% of their nutritional requirements at the end of the stay, which was calculated by monitoring food intake and oral nutritional supplement consumption. The *process indicator* chosen was the time interval between hospital admission and surgery (hours) and the *financial indicator* was based on the costs related to the number of days spent in hospital (days).

### Statistical analysis

Parametric tests and non-parametric tests were used for comparison of length of stay (LOS) (Student, Wilcoxon, Trend tests). The chi-squared test was used for dichotomous variables. Descriptive analysis was completed when data were not available before the onset of the CP.

## Results

### Patients

From March to December 2011, 233 patients were considered suitable for initial inclusion in this CP. One hundred fifty-nine patients completed their entire hospital stay within the CP. The main reasons for exclusion were a transfer to another department where the CP was not implemented, death, conservative treatments, pathological fractures, and cases where the time interval between emergency admission and surgery exceeded 72 h (details in Table 1). In 2011, the rate of exclusion was 31.7%. In view of these figures, it was decided to open the CP across more units (01.05.2012) and to include cases with hospital admission to surgery time intervals of more than 72 h. The exclusion rate dropped to 17.4% in 2012. In 2012, 310 patients were initially included in this CP after emergency unit admission, 256 of whom remained in the CP for their entire hospital stay. In 2013, we definitively included 254 patients in this CP with a lower exclusion rate of 9.9%. From 01.03.11 to 31.12.13, the application of these criteria yielded an ultimate total of 669 patients who had followed this CP.

**Table 1:** **Exclusion criteria analysis**.

Criteria	2011	2012	2013
	
	Number of patients		
Transfer to another unit	53	21	7
Deceased	6	9	13
Treated without surgery	5	6	3
Intensive care	4	7	0
Pathological fracture, amputation	3	6	2
Operated within 72 h	3	3	0
Subtrochanteric fracture		1	2
Fracture without fall			1
Attribution error		1	
**Total**	**74**	**54**	**28**
**Percentage**	**31.7%**	**17.4%**	**9.9%**

### Clinical indicators

The first clinical indicator was the prevalence of delirium on the third post-operative day (D3). In 2012, 83.6% of patients were evaluated, 16.3% of whom were found to have a positive CAM at D3. In 2013, 56% of patients were evaluated at D3 and 12.7% of these patients had delirium. The second clinical indicator, the rate of occurrence of pneumonia remained stable at 3.14 and 3.12% for the first 2 years and then decreased to 2.75% in 2013.The third clinical indicator, the proportion of patients fulfilling at least 75% of their nutritional needs at the end of the hospitalization period, increased from 37 to 60% between 2011 and 2013.

### Process indicators

**Figure 2:**
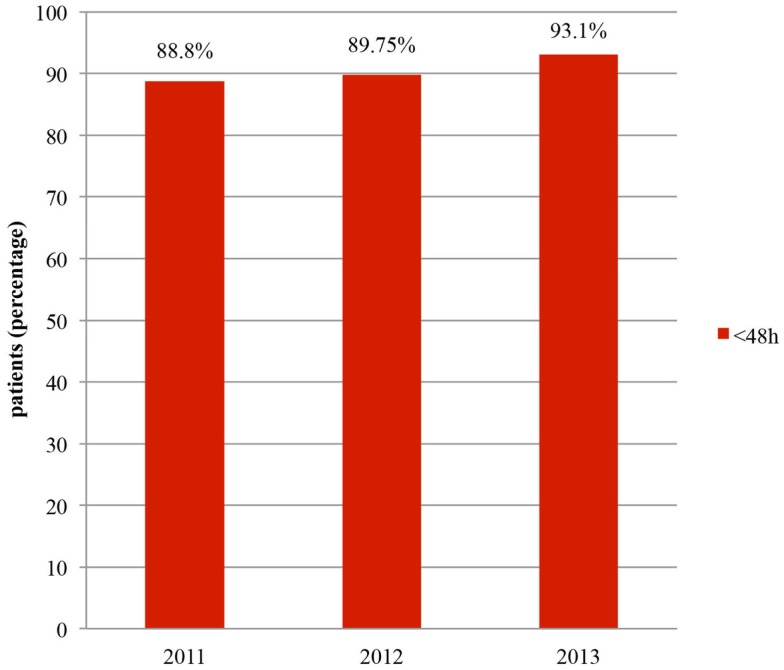
**Surgery within 48 h**.

**Figure 3:**
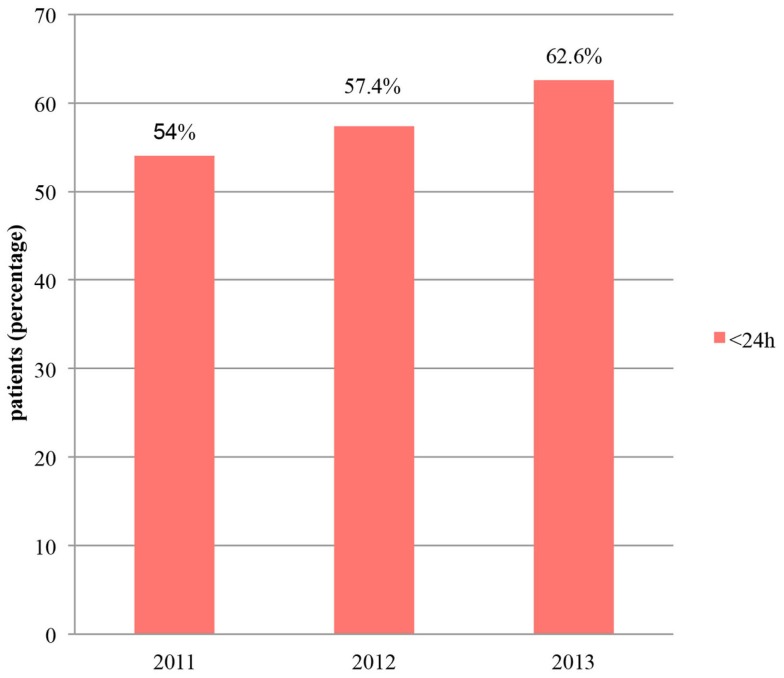
**Surgery within 24 h**.

In terms of the time interval between emergency admission and surgery, the goal of the CP was to ensure that at least 90% of patients underwent surgery within 48 h. At the end of 2011, 88.8% of patients were treated within 48 h (Figure 2). In 2012, this figure dropped to 85.5%, due, in part, to the inclusion of patients who had an extended pre-operative period of longer than 72 h. However, if we excluded the patients who waited for medical reasons (cardiac or neurologic investigations, etc.), and therefore non-structural reasons (evaluated in 12 patients), this rate was then better than 2011, with a value of 89.75%. In 2013, with the same approach, 93.1% of patients underwent surgery within 48 h and 62.6% within 24 h (Figure 3).

### Financial indicators

**Figure 4:**
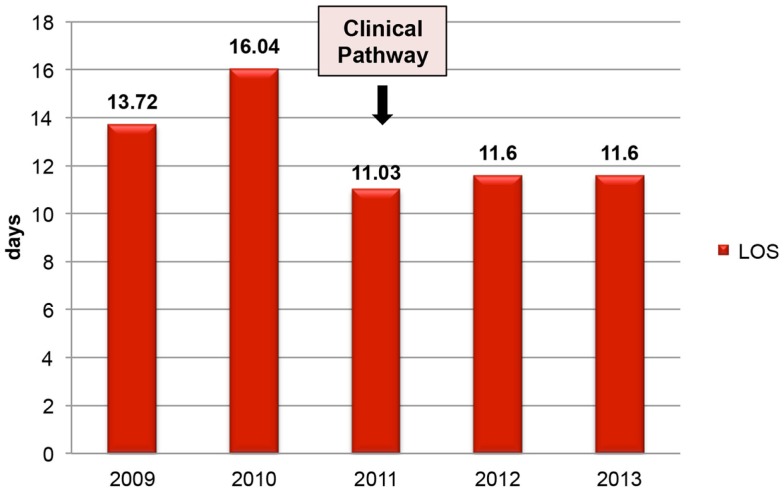
**Length of stay in acute care (days)**.

The financial indicator chosen was the average LOS in hospital. As shown in Figure 4, fractures of the proximal femur, without major complications, accounted for 16 days in 2010 before the introduction of the CP. After initiation of the CP, this figure dropped to 11 days and remained constant thereafter (*p* Student <0.001, *p* Wilcoxon <0.001, *p* Trend test <0.001) (Figure 4).

We noted that in 2012, 52% of the patients went to a rehabilitation center at the end of their stay in acute care hospital, 32% to a nursing home, and <10% went directly home. In 2013, these figures were 55.1, 26.8, and 10.2%, respectively. In 2013, 35.4% of these patients were transferred to their previous living place. We observed an increase of patients going back home with a trend test almost significant (*p* = 0.57) and a chi-squared test that is statistically significant between 2010 and 2012 (*p* = 0.002).

## Discussion

With this femoral fracture CP, we targeted a model of care, which focused on the patients’ needs, through a multidisciplinary approach. The aim was to combine a better quality of care for the patient, with a reduction in hospitalization costs. This CP was made possible with the concerted efforts of emergency physicians, orthopedic surgeons, nurses, unit management, responsible for the flow of patients through the system, physiotherapists, geriatricians, specialists in osteoporosis, and nutritionists. The osteoporosis specific treatment, which is given after the fracture for these patients, was not evaluated here as it is part of another CP (osteoporosis CP). After 3 years, we noted significant improvements in terms of the number of patients included in the CP (almost 90%).

Concerning the first clinical indicator, the prevalence of delirium, evaluations showed a decrease in the rate of positive delirium diagnosis in 2 years. This emphasized the importance of sharing knowledge between geriatricians and other medical personnel. This was particularly important in terms of necessity of frequent CAM assessments: this allowed to identify, treat, and prevent incidences of delirium. Within this program, in 3 years, we noted a clinically significant improvement in the rates of positive delirium detection by the nursing staff. However, this did not negate the essential role of geriatricians in the pre-operative and post-operative care of these patients (12). As a comparison in Brisbane (13), 54% of the patients with hip fractures experienced post-operative delirium. A rate of 12.7% was observed here after the implementation of the CP.

The meta-analysis of CPs for hip fracture cases of Neuman (8) found no significant differences in the proportion of post-operative pneumonia diagnoses, between pathway patients who underwent surgery within 36 h of hospital admission, and those receiving usual care. In our hospital, no statistical difference in this rate after 3 years of the CP was also found. However, early surgery, within 48 h of hospitalization, should reduce the risk of pneumonia among patients according to the systematic review and meta-analysis of Simunovic (14).

One of the goals of the CP was to have 100% of patients fulfilling at least 75% of their nutritional needs at the end of hospitalization. Three years after CP implementation the results had improved but, it still remains a weak point of this program. Indeed, two main barriers were observed. The first was missing or incomplete data on caloric intake. Subsequently, joint efforts by the Clinical Nutrition Team and the Orthopedic unit sought to increase the number of regular assessments. The second barrier was the loss of patients appetite and the difficulty of increasing food intake at mealtimes, despite support from the medical team. A strategy to encourage the consumption of snacks between meals has been proposed to overcome this issue. Though, this will require re-organization of the nursing day care. A recent study has shown that patients with higher caloric intake have lower complication rates and a shorter hospital stay on average (15).

An important clinical improvement after the implementation of the CP was the increase in the number of patients undergoing surgery within 48 h of admission. This rate was 88.8% after the first year of CP in 2011 and had increased to 93.1% by 2013. Particular efforts by surgeons to reduce the pre-operative period contributed massively to these excellent results. In comparison, the United Kingdom National Hip Fracture Database (UKD) (7) reported that the percentage of patients, with complete data, treated within 48 h of admission and within normal working hours had risen from 80% in 2010 to 87% in 2011, 83% in 2012, and 86% in 2013 (all patients who were medically unfit on admission were excluded). Similar results were found in 2009 in Hong-Kong, with 68% of the patients with hip fractures who underwent surgery within 48 h of hospital admission; in the hospital where a CP had been implemented since 2007, this rate was 86% (16).

The average LOS in our hospital for proximal femur fracture patients, without major complications, decreased from 16 days in 2010 to 11 days in 2012 and 2013. In UK (7), the national reports showed a mean length of acute stay of 16.4 days in 2011 and 15.7 days in 2013, which compare favorably with the average of 19.7 days observed in 2010. Another experience in Hong-Kong (16) showed that, after CP implementation the LOS in acute hospital reduced from 12.07 days in 2006 to 8.27 days in 2007, 7.67 days in 2008, and 6.66 days in 2009. However, some units provide both acute and rehabilitation services while other units favor quick discharge. Therefore, though we observed a decrease in the LOS in each hospital, which had a CP in operation, the LOS itself was dependent of individual hospital policy.

Finally, the communication between numerous clinicians has increased and is now perpetuated through regular CP review meetings. The successful deployment of a CP demanded concerted action and effective cooperation between different health professionals. We deem it essential that, as we did from initiation, a project manager is in place to ensure adequate data collection, provide training for the various care teams and oversee meeting coordination and communication between various disciplines. This is to safeguard the efficiency of the CP, and reassess aspects of the CP, with all partners, if required. Where necessary, responsibilities may also extend to the issuing of warnings when deficiencies in the process are identified. According to the Hong-Kong CP study also, an orthopedic nurse was engaged as project manager, and was responsible for data collection and auditing, and was considered to be a key element in the successful execution of the scheme (16).

## Conclusion

Implementation of a CP for proximal femoral fractures had a positive impact. This CP facilitated an improvement in the qualitative and quantitative efficiency of proximal femoral fracture management in elderly patients particularly in terms of clinical, process, and financial factors. These good results should allow people to extend the concept of CP to patients with other common musculoskeletal pathologies.

## Conflict of Interest Statement

The authors declare that the research was conducted in the absence of any commercial or financial relationships that could be construed as a potential conflict of interest.
